# Phospho-Tau Signature During Mitosis: AT8, p-T217 and p-S422 as Key Phospho-Epitopes

**DOI:** 10.3390/cells14201638

**Published:** 2025-10-21

**Authors:** Marion Goussard, Kelly Zarka, Morgane Denus, Thomas Curel, Sylvie Claeysen, Bruno Lefebvre, Malika Hamdane, Philippe Marin, Julien Villeneuve, Marie-Laure Parmentier

**Affiliations:** 1 IGF, Univ Montpellier, CNRS, Inserm, 34094 Montpellier, Francesylvie.claeysen@igf.cnrs.fr (S.C.); philippe.marin@igf.cnrs.fr (P.M.); julien.villeneuve@igf.cnrs.fr (J.V.); 2 Inserm, CHU Lille, CNRS, LilNCog—Lille Neuroscience & Cognition, University of Lille, 59000 Lille, France; bruno.lefebvre@inserm.fr (B.L.); malika.hamdane@inserm.fr (M.H.)

**Keywords:** tauopathy, mitosis, biomarker, Alzheimer’s disease

## Abstract

Tau was initially identified as a microtubule-binding protein critical for microtubule stabilization. It is also a pathological hallmark of tauopathies, a group of neurodegenerative diseases that include Alzheimer’s disease. Under pathological conditions, Tau becomes hyperphosphorylated at numerous sites and aggregates into filamentous deposits, contributing to neuronal cell death and disease progression. While significant research has focused on Tau phosphorylation dynamics and their consequences in pathological contexts, comparatively few studies have investigated Tau phosphorylation during physiological processes, despite the potential relevance to the early onset of pathology. Previous findings have suggested similarities between mitotic Tau phosphorylation and hyperphosphorylation observed in tauopathies, particularly at sites such as AT8, PHF1, S214, and S422. In this study, we quantified the relative levels of phosphorylation at 12 Tau phospho-epitopes during interphase and mitosis in vitro to establish a preliminary mitotic phospho-Tau signature, which was subsequently validated in vivo. Our results demonstrated pronounced phosphorylation of Tau at AT8, p-T217, and p-S422 epitopes during mitosis, both in vitro and in vivo. These findings provide new insights into the physiological phosphorylation of Tau and its potential links to pathological processes.

## 1. Introduction

Tau is primarily known as a microtubule-associated protein that stabilizes microtubules in neurons [1,2]. Phosphorylation of Tau was reported to be associated with a reduced affinity in microtubule binding [3]. This effect is particularly pronounced at phosphorylation sites within the microtubule binding domain, such as S262 and S356 [4,5,6], as well as in other regions. For example, phosphorylation at S214 within the proline-rich region [7,8] and at S396 and S404 in the C-terminal domain—phospho-residues that together constitute the PHF1 epitope—also impairs microtubule binding [9,10].

Many of these phosphorylation sites are found to be hyperphosphorylated in the context of neurodegenerative disorders like Alzheimer’s disease (AD) [11]. This is considered to contribute to Tau aggregation into intracellular Tau filamentous deposits, a process associated with neuronal cell death and the emergence of AD [12,13,14]. Specific phosphorylation events are linked to distinct stages of the disease [15]. For instance, the AT8 epitope, involving phosphorylation at S202 and T205, is frequently detected in pre-tangle neurons, marking early AD pathology [16,17]. Among emerging biomarkers, phosphorylation of Tau at T217 has garnered attention for its utility in early-stage tauopathy diagnosis. It was recently shown to outperform the measure of phosphorylation at T181 as a cerebrospinal fluid (CSF) and blood biomarker for AD [18,19,20,21]. Similarly, phosphorylation at S422 is considered a marker for early disease events in both AD and other tauopathies [22,23].

Microtubule dynamics are essential for spindle assembly, chromosome alignment, and segregation during mitosis. Since Tau is expressed not only in the nervous system but also in other cell types—such as lung epithelial cells, pancreatic acinar cells, enteroendocrine cells, and cancer cells [24,25,26,27,28,29]—understanding Tau phosphorylation during mitosis is of significant interest. Previous studies have revealed notable similarities between Tau phosphorylation patterns observed during mitosis and in AD, particularly at phosphorylation epitopes AT8, PHF1, p-S214 and p-S422 [7,30,31,32,33,34].

Specifically, phosphorylation of Tau at PHF1 and AT8 epitopes during mitosis was initially identified in cell lines stably expressing Tau [34] and in neuroblastoma cell lines synchronized with nocodazole treatment [33,35]. Mass spectrometry analysis of Tau peptides in artificially synchronized cells further suggested that S214 phosphorylation occurs during cell division [7]. Additionally, studies using Xenopus meiosis and neuroblastoma cell lines demonstrated Tau phosphorylation at S422 during mitosis [31].

In this study, we aimed to characterize Tau phosphorylation sites specifically enriched during mitosis compared to interphase under conditions of transient Tau expression, without the use of microtubule-depolymerizing drugs. To this end, we quantified the relative levels of phosphorylation at 12 Tau phospho-epitopes during interphase and mitosis within the same biological system to establish a preliminary Tau phospho-signature during mitosis. To circumvent the limitations of continuous Tau overexpression, we utilized an SHSY-5Y cell line with inducible Tau expression. Immunocytochemistry experiments, featuring co-labeling of dividing cells, were performed to avoid artificial mitotic arrest with microtubule polymerizing or depolymerizing agents. Finally, we validated this mitotic Tau phospho-signature in vivo using dividing epithelial cells of *Drosophila melanogaster* overexpressing human Tau. Our results revealed strong phosphorylation of Tau during mitosis at AT8, p-T217, and p-S422 epitopes both in vitro and in vivo. These findings offer new perspectives on the intersection of fundamental biology focusing on cell division and the molecular mechanisms underlying Tau-associated diseases.

## 2. Material and Methods

### 2.1. Cell Culture

Tau-inducible cell line was established from the neuroblastoma cell line SH-SY5Y by using the Tet-on T-rex system [36]. Cells were grown in complete culture medium consisting of Dulbecco’s modified Eagle’s medium/F-12 medium supplemented with 10% heat-inactivated FBS (Gibco, ThermoFisher Scientific, Waltham, MA, USA, #10270-106), 50 U/mL penicillin/streptomycin, and 2 mM L-glutamine (Sigma-Aldrich, St. Louis, MO, USA, #G7513) and maintained in humidified atmosphere containing 5% CO_2_ at 37 °C. Selection was maintained with 5 µg/mL blasticidin and 100 µg/mL Zeocin. Induction of Tau expression (1N4R isoform) was performed for 24 h with 1 µg/mL tetracyclin 24 h after having plated the cells in 24-well plates for immunocytochemistry experiments [32]. At this time point, increased Tau expression is observed [36] with no significant disruption of mitosis detected.

### 2.2. Fly Stocks

We used the ptc-Gal4 activator strains as in Bougé and Parmentier [37]. The UAS-hTau^WT^ strain was a gift from Mel Feany [38]. Third instar stage larvae were used independently of their sex.

### 2.3. Immunocytochemistry

Cells grown on coverslips were fixed with 4% paraformaldehyde for 5 min at room temperature or. Cells were then permeabilized with PBS 1×, Triton 0.3% (PBS-T), at room temperature and then incubated with blocking buffer (PBS-T, 0.3% BSA) for 30 min at room temperature. Cells were then incubated with primary antibodies diluted in blocking buffer for 1 h at room temperature, followed by PBS-T wash and incubation with secondary antibodies.

Third instar larval imaginal discs were dissected in PBS 1× and fixed for 20 min in 4% paraformaldehyde. After permeabilization with PBS 1×, Triton 0.3% (PBS-T) for 30 min at room temperature, discs were incubated overnight at 4 °C with primary antibodies diluted in PBS-T, 0.3% BSA. Fluorescent secondary antibodies were used at the recommended dilution and incubated for 1 h at room temperature with 300 nM DAPI to counterstain nuclei, when needed.

Preparations were mounted using Dako fluorescent mounting medium (Agilent Technologies, Inc., Santa Clara, CA, USA, Cat. # S3023).

### 2.4. Antibodies

Primary antibodies were: Pan-Tau antibodies: rabbit polyclonal anti-Tau (Dako, Agilent Technologies, Inc, Santa Clara, CA, USA, #A002401, 1:2000) and mouse anti-Tau monoclonal T46 (ThermoFisher Scientific, Waltham, MA, USA, Cat. #13-6400, 1:200), mouse monoclonal anti-GFP (Roche, Basel, Switzerland, Cat. #11814460001, 1:5000), sheep polyclonal anti-tubulin (ATN02, Cytoskeleton Inc., Denver, CO, USA, 1:400), rat monoclonal anti-tubulin (CBL270, Merck KGaA, Darmstadt, Germany, 1:300), mouse anti-PH3 (phospho-Ser10, clone 3H10, 1:300), rabbit anti-PH3 (phospho-Ser10 + Thr11, Cat. ab32107, Abcam, Cambridge, UK, 1:1000). Anti phospho-Tau antibodies used in this study are listed in the table below.
**Name of Antibody or Phospho-Epitope****Reference****Provider****Dilution**AT8MN1020Invitrogen (ThermoFisher Scientific) Waltham, MA, USA)1:200S202[EPR2402] ab108387Abcam, Cambridge, U.K.1:200T20544-738 GInvitrogen 1:200PHF1Gift from P. Davies
1:500S396[EPR2731] ab109390Abcam1:200S404[EPR2605] ab92676Abcam1:200AT100MN1060Invitrogen1:200T21244-740 GInvitrogen1:200S21444-742 GInvitrogen1:200T21744-744Invitrogen1:200S416
Cell Signaling Technologies, Danvers, MA, USA1:200D7U2P (#15013) S422[EPR2866] ab79415Abcam1:200

Secondary antibodies were Alexa-Fluor-488, Alexa-Fluor-633 (ThermoFischer Scientific, Waltham, MA, USA), Cy3 and Cy5 (Jackson ImmunoResearch, Westgrove, PA, USA), all diluted between 1:1000 and 1:500.

### 2.5. Confocal Microscopy

Images were acquired using a LSM780 and LSM980 laser confocal laser scanning microscope with 20× Plan Apo 0.8NA and 40× Plan Apo oil 1.3NA objectives at the imaging facility MRI (Biocampus, UM-CNRS-INSERM, Montpellier, France). For adequate measurements and comparison of immunoreactivity intensities, confocal laser settings were set up to avoid saturation of signal and were not modified when measuring different conditions of a specific staining. After acquisition, images were processed with Fiji [39]. Images displayed in the figures are maximal intensity z-projections of 4 to 8 z-stack images with minor brightness adjustments to ensure consistent rendering across confocal sessions.

### 2.6. Quantification and Statistics

Quantification of the level of Tau and phospho-Tau immunoreactivity was made as follows: dividing cells were selected based on the mitotic spindle visible with the tubulin staining independently of the other stainings, and a cytoplasmic area was selected around the mitotic spindle to measure total Tau and/or phospho-Tau staining (mean intensity within the selected area). Interphasic cells were chosen nearby measured mitotic cells for quantification of interphasic Tau and phospho-Tau stainings. In addition to qualitative observations, two to three biological replicates were dedicated to quantification, with a minimum of three images per condition (±tetracycline). Each condition included 12–24 quantified cells. Figures display the results of a representative experiment, alongside a selected image area for illustration.

The Principal Component Analysis (PCA) was conducted using eigenvalues greater than 1, without applying a rotation method. Components 1 and 2 accounted for 53.98% and 39.47% of the variance, respectively. The analysis was performed using Jamovi software (2024, Version 2.6; www.jamovi.org retrieved on 3 March 2025), and graphical representation was generated using the snowCluster Jamovi module (Version 7.4.8; github.com/hyunsooseol/snowCluster retrieved on 24 September 2025).

For in vivo analyses, at least three wing discs from Tau-overexpressing Drosophila were examined for each phospho-epitope. Each experiment was replicated three times to ensure reproducibility.

## 3. Results

### 3.1. Tau Phosphorylation Pattern During Mitosis in Tau-Inducible SH-SY5Y Cell Line

**Figure 1:**
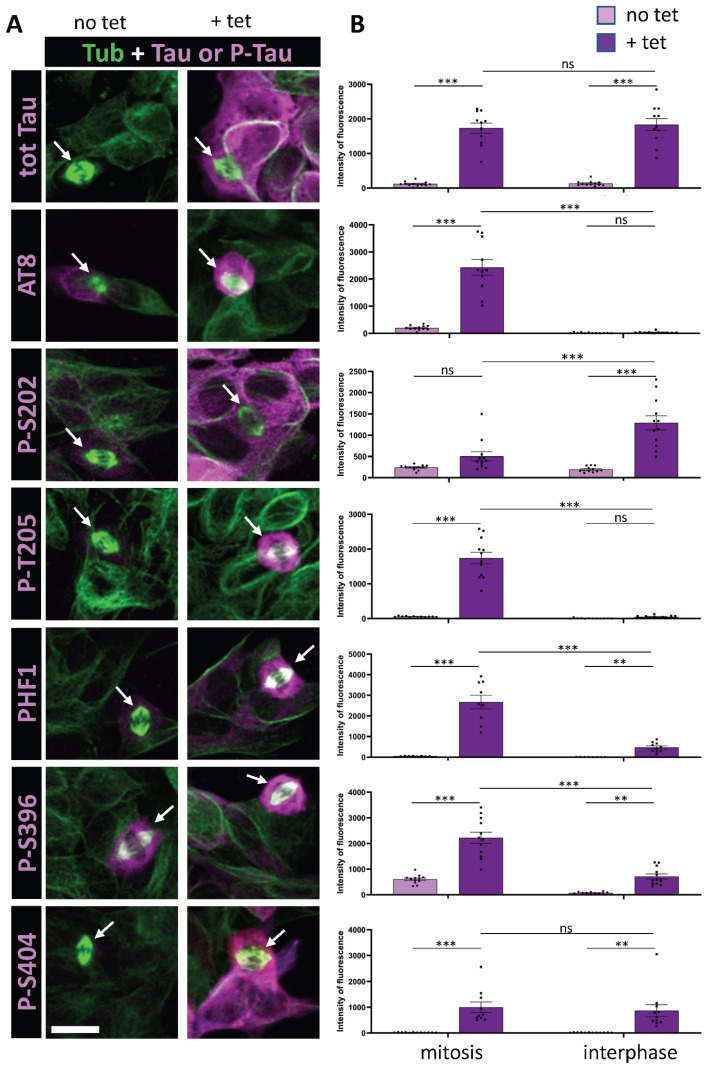
Immunostaining of AT8 and PHF1 Tau phospho-epitopes in Tau-inducible SH-SY5Y cell line. (**A**) Confocal images of interphase and mitotic cells with or without tetracycline-induced Tau expression. Tubulin staining (green) highlights the mitotic spindle in dividing cells (arrows). Total Tau or phospho-Tau staining is shown in magenta. Image dimensions: 40–50 µm^2^. (**B**) Quantification of a representative experiment for each phospho-epitope (data available in Supplementary Table S2), showing the intensity levels of total Tau or phospho-Tau staining in mitotic (left) and interphase cells (right), with (+tet) and without (no tet) Tau induction. The top panel demonstrates comparable total Tau staining levels during mitosis and interphase upon tetracycline induction. The AT8 epitope shows no staining increase in interphase upon induction but exhibits a marked increase in mitotic cells. For p-T205, PHF1, and p-S396, staining increases in interphase with an additional rise in mitotic cells. In contrast, p-S202 and p-S404 exhibit increased staining in interphase upon Tau induction, without further enhancement in mitotic cells. Kruskal–Wallis ANOVA was performed to analyze significance of differences between groups for each experiment: ** *p* < 0.01; *** *p* < 0.001, ns (non significant). Scale bar is 15 µm.

**Figure 2:**
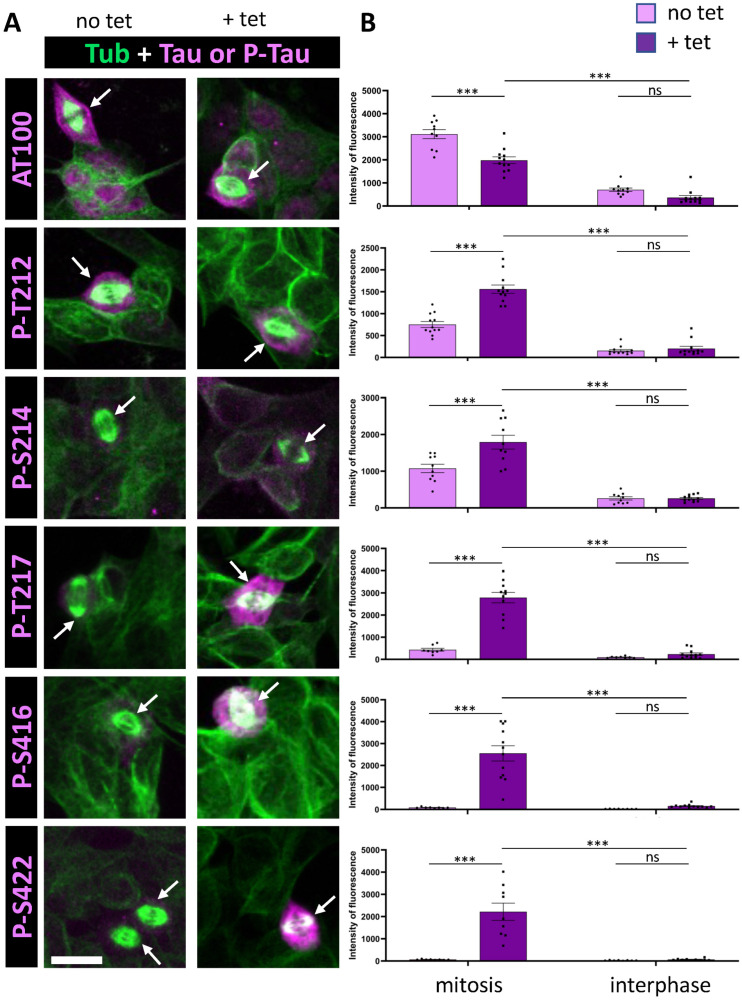
Immunostaining of AT100, p-S416, and p-S422 Tau phospho-epitopes in Tau-inducible SH-SY5Y cell line. (**A**) Confocal images of interphase and mitotic cells with or without tetracycline-induced Tau expression. Tubulin staining (green) highlights the mitotic spindle in dividing cells, and phospho-Tau staining is shown in magenta. Image dimensions: 40–50 µm^2^. (**B**) Quantification of a representative experiment for each phospho-epitope (data available in Supplementary Table S2), showing the intensity levels of phospho-Tau staining in mitotic (left) and interphase cells (right), with (+tet) and without (no tet) Tau induction. For AT100, p-T212, and p-S214, minimal staining increases in interphase are observed upon induction, along with less than a 2.5-fold increase in mitotic cells. Conversely, p-T217, p-S416, and p-S422 show little to no increase in interphase staining but display a marked increase in mitotic cells upon Tau induction. Kruskal–Wallis ANOVA was performed to analyze significance of differences between groups for each experiment: *** *p* < 0.001, ns (non significant). Scale bar is 15 µm.

**Figure 3:**
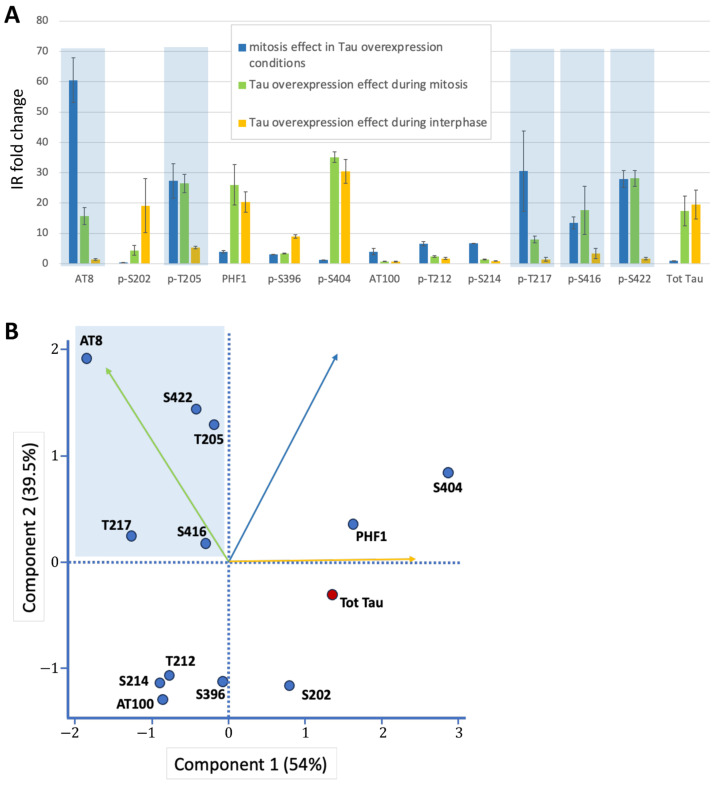
Graphical representations of Tau phosphorylation changes. (**A**) Fold-change analysis of immunoreactivity (IR) across three conditions: (i) Mitosis vs. interphase under Tau overexpression. (ii) Tau overexpression during mitosis (vs. baseline). (iii) Tau overexpression during interphase (vs. baseline). Mitosis-specific phospho-epitopes (high fold-changes in i and ii, low in iii) are highlighted in blue. (**B**) PCA plot of Tau epitopes along the first two principal components. The mitotic phospho-epitope cluster (blue) is characterized by high values for Component 2 (driven by mitosis-related variables i and ii) and low values for Component 1 (primarily influenced by interphase overexpression effects, variable iii). Variable contributions to each component are indicated by vectors (color-coded as in panel A). To distinguish it from other phospho-epitopes (blue dots), total Tau staining is represented by a red dot.

We utilized the SH-SY5Y neuroblastoma cell line transfected with Tau 1N4R transgene, regulated by the tetracycline-controlled T-Rex mammalian expression system [32,36]. This approach enabled the investigation of Tau phosphorylation under two conditions: (1) low basal expression levels, and (2) a 20-fold increase in Tau expression following 24-h induction with tetracycline (Figure 1 and Figure 2, Table 1). To examine phosphorylation under normal cell cycling conditions, we did not arrest the cell cycle. Instead, we performed immunocytochemistry on fixed cells, co-labeling for microtubules to identify mitotic spindles. Total Tau or phospho-Tau labeling intensity was quantified in the cytoplasm of interphase and mitotic cells under both basal and Tau overexpression conditions. This allowed us to determine which phospho-epitopes exhibited the greatest increase in staining during mitosis relative to interphase under conditions of Tau overexpression (Table 1, Figure 3A). Hence, the first selection criteria were the degree of increase in phospho-epitope immunoreactivity between interphase and mitosis in conditions of Tau overexpression, indicating mitosis-specific staining. As a control, total Tau staining revealed no significant change in staining between interphase and mitosis in conditions of Tau overexpression (Table 1). The second selection criteria were the magnitude of the increase in phospho-epitope immunoreactivity during mitosis between basal and Tau-overexpression conditions, reflecting specificity for Tau staining (Table 1, Figure 3A).

We initially focused on the previously characterized phospho-epitopes AT8 and PHF1 along with their associated phosphorylation sites: S202 and T205 for AT8, and S396 and S404 for PHF1. AT8 epitope was previously shown to be strongly phosphorylated during mitosis, whereas the increase in phosphorylation at PHF1 site was only partial [32,35]. Our findings revealed a 60-fold increase in AT8 staining specifically during mitosis under conditions of Tau overexpression, alongside a 16-fold increase in AT8 staining in mitotic cells when comparing basal and Tau-overexpression conditions (Figure 1 and Table 1). This indicates that Tau undergoes specific phosphorylation at this epitope during mitosis. In contrast, PHF1 displayed only 4-fold increase in immunoreactivity in mitotic cells compared to interphase cells under Tau overexpression conditions. This was accompanied by a marked 20-fold increase in staining associated with Tau overexpression during interphase (Figure 1 and Table 1). These findings suggest that phosphorylation at the PHF-1 epitope predominantly occurs during interphase under conditions of Tau overexpression. Altogether, our results are in accordance with previously reported data on the AT8 and PHF1 epitopes during mitosis. Analysis of individual phosphorylation sites revealed that mitosis-specific phosphorylation is associated with T205 for AT8 and S396 for PHF1, showing a 27- and 3-fold increase in immunoreactivity during mitosis under Tau overexpression conditions, respectively. In contrast, S202 and S404 displayed a pattern of increased phosphorylation primarily during interphase in response to elevated Tau levels, with 19- and 30-fold increases, respectively (Figure 1 and Table 1).

We then analyzed a series of additional phospho-epitopes, including AT100, p-S214 and p-S422, all previously reported to be phosphorylated during mitosis [7,31,32,35]. We examined the phosphorylation sites T212 and T217, which are components of the AT100 epitope along with S214 [40], and the recently characterized S416 as an additional distal phosphorylation site [41]. Under conditions of Tau overexpression, our analysis revealed a modest increase in staining for AT100 and S214 during mitosis (4-fold and 7-fold, respectively); however, there was no clear increase in staining of mitotic cells between basal and Tau overexpression conditions (0.75-fold and 1.42-fold, respectively) (Figure 2, Table 1). This suggests an absence of specific Tau phosphorylation at these epitopes. For the other phosphorylation sites within the AT100 epitope, both T212 and T217 showed increased phosphorylation during mitosis under conditions of Tau overexpression, with T217 showing a much stronger response (6.6-fold and 30-fold, respectively). Additionally, the distal phospho-epitopes p-S416 and p-S422 demonstrated pronounced increases in staining during mitosis, with 13-fold and 28-fold increases in immunoreactivity under Tau overexpression conditions, respectively (Figure 2, Table 1).

Overall, Principal Component Analysis using the three variables from Table 1 identified the mitotic phospho-epitope cluster, characterized by high values for Component 2 (driven by the two mitosis-related variables) and low values for Component 1 (primarily influenced by interphase overexpression effects) (Figure 3B). This cluster contains AT8, p-T205 (part of the AT8 epitope), p-T217, p-S416, and p-S422 as Tau epitopes highly and specifically phosphorylated during mitosis (Figure 3A,B).

Having identified this signature under normal cell cycle conditions in a neuroblastoma cell line, we assessed its generality and robustness in vitro, by extending our analysis to (i) a distinct cell line: HeLa cells stably expressing the 0N4R Tau construct [42], (ii) an alternative technique: Western blot analysis, which required cell cycle synchronization. Our results in HeLa cells confirmed the mitotic phosphorylation of these epitopes (Supplementary Figure S1), thereby validating the signature across in vitro experimental systems.

### 3.2. AT8, p-T217 and p-S422 Phospho-Epitopes Are Specifically Associated with Mitotic Cells Under Conditions of Tau Overexpression In Vivo

To further evaluate the relevance of mitotic phosphorylation at the specific phospho-epitopes identified in cell culture, we analyzed their behavior in vivo under conditions of human Tau overexpression. For this purpose, we used a *Drosophila* transgenic system that overexpresses human 0N4R Tau within a determined area of the epithelial wing disc during larval development, as described in Bougé and Parmentier [37]. The tissue undergoes growth during development, with all cells undergoing mitosis. Mitotic cells were identified using the phospho-histone 3 (PH3) marker.

**Figure 4:**
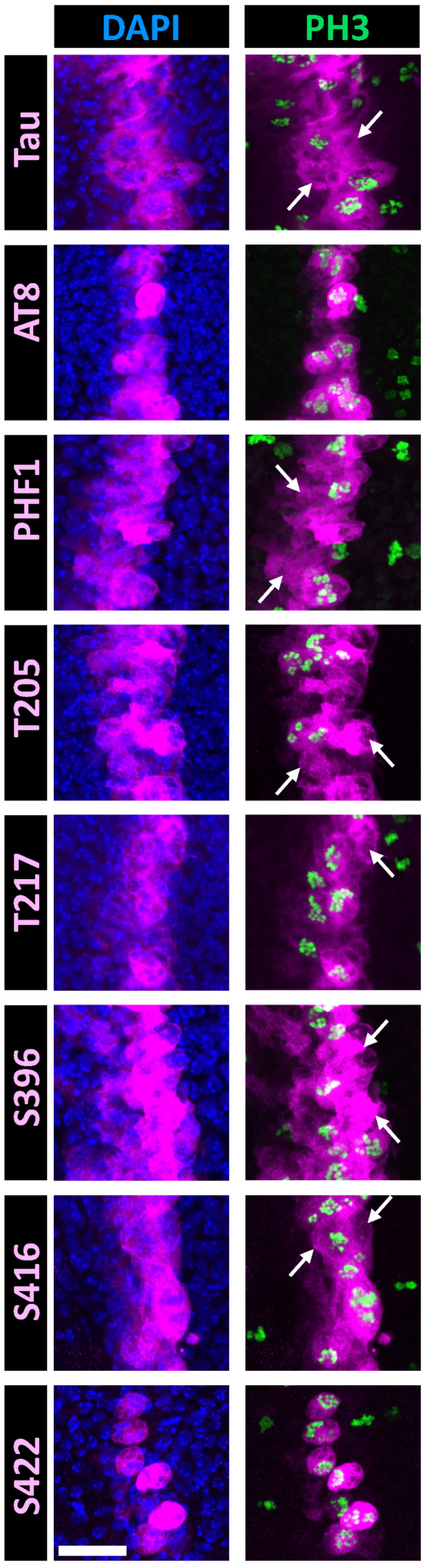
Double immunostaining of selected Tau phospho-epitopes with PH3 in the *Drosophila* wing disc at the third instar larval stage. Confocal images of a band of cells overexpressing human hTau in the Drosophila wing disc. Staining for total Tau is shown in the upper row (magenta), while staining for Tau phospho-epitopes is displayed in the lower rows (magenta). Nuclei are labeled with DAPI (blue, left column), and mitotic cells are identified by PH3 labeling (green, right column). Total Tau staining reveals that hTau is expressed within a 3–4 cell-wide band in the wing disc, consistent with the ptc-Gal4 expression pattern at this developmental stage. Since mitosis occurs asynchronously throughout the disc, PH3-positive mitotic cells are visible both within and outside the Tau-overexpressing region. In this analysis, we focused on the Tau-overexpressing area to examine the concordance between PH3 and phospho-Tau labeling. Arrows indicate interphase cells labeled for total Tau or phospho-Tau. Numerous interphase cells are observed for PHF1, p-T205, and p-S416 staining; fewer are detected for p-T217; and none are present for AT8 or p-S422 staining. Scale bar is 20 µm.

**Figure 5:**
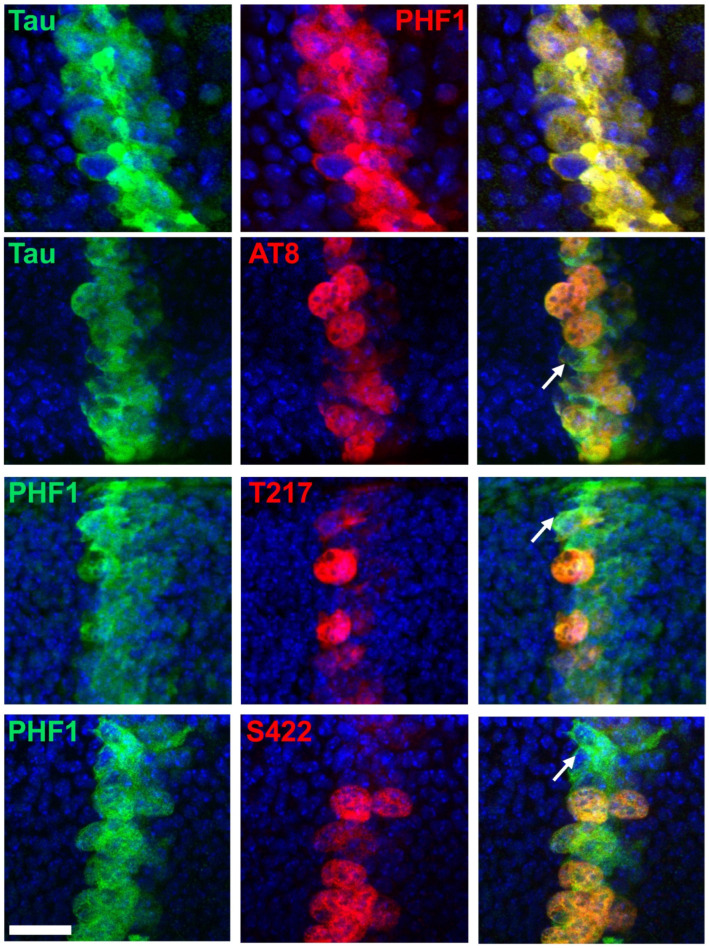
Double immunostaining of selected Tau phospho-epitopes with total Tau in the *Drosophila* wing disc at the third instar larval stage. Confocal images of the band of cells overexpressing human hTau, showing staining for total Tau (green), Tau phospho-epitopes (red), and merged images (right column). PHF1 (red in top images, green in bottom images) staining colocalizes strongly with total Tau staining, while AT8, p-T217, and p-S422 label only a subset of cells expressing human Tau. Arrows highlight cells positive for total Tau but negative for phospho-Tau. Scale bar is 20 µm.

Consistent with previous findings, PHF1 and p-S396 epitopes were detected throughout the entire domain of Tau overexpression (Figure 4), encompassing both mitotic and non-mitotic cells. Double immunostaining confirmed the presence of PHF1 in all Tau-overexpressing cells (Figure 5). Similarly to previous findings, we could not detect any specific signal associated with epithelial Tau-expressing cells for p-S214 and AT100 epitopes [43].

When assessing the phospho-epitopes identified in cell-culture experiments, we observed that immunoreactivity for AT8, p-T217, and p-S422 was predominantly restricted to mitotic cells. In contrast, immunoreactivity for p-T205 and p-S416 was also present in many non-mitotic cells (Figure 4). Double staining with either PHF1 or total Tau further confirmed that AT8, p-T217, and p-S422 epitopes were restricted to a subset of Tau-expressing cells (Figure 5). These results validate the mitotic specificity of AT8, p-T217, and p-S422 epitopes.

## 4. Discussion

The use of an inducible Tau expression system to study Tau phosphorylation during mitosis has proven highly beneficial in multiple aspects. First, by comparing Tau phosphorylation levels before and after a 24-h transient induction, we could identify specific phosphorylation sites that were prominently phosphorylated during interphase under Tau overexpression conditions. These included PHF1, S202, S404, and, to a lesser extent, S396. These findings are internally consistent, as p-S396 and p-S404 together constitute the PHF1 epitope. Moreover, our results align with prior studies comparing Tau phosphorylation in the LAN-5 cell line (endogenous levels of Tau) and a CHO cell line with stable Tau overexpression. Those studies similarly demonstrated high phosphorylation levels at S202 and S404 under conditions of stable overexpression of Tau [7]. Furthermore, these observations provide a plausible explanation for variations in mitosis-specific phosphorylation of the PHF1 epitope across different cell lines, as these variations likely depend on the baseline phosphorylation levels during interphase [33,35].

Second, by avoiding the use of microtubule polymerizing or depolymerizing drugs to arrest cells in M phase, we minimized potential indirect effects of these agents. This approach may explain some discrepancies between our findings and previously published data. For instance, we did not observe p-S214 as a mitosis-specific phospho-epitope, as reported by Illenberger et al. [7], who used nocodazole-treated cells. Similarly, we did not identify AT100 as a mitosis-specific phospho-epitope, contrary to the findings of Delobel et al. [31], who studied progesterone-induced maturation of Xenopus oocytes and Tau-stably transfected neuroblastoma cells. Given the strong Tau-specific staining we achieved with both p-S214 and AT100 antibodies under the same immunocytochemistry conditions on specific *Drosophila* tissues [43], we can exclude antibody inefficiency as a contributing factor. Finally, among the tested phospho-epitopes, we identified novel mitosis-specific phosphorylation sites, which are p-T205, p-T217 and p-S416. Additionally, we confirmed previously described mitosis-specific phosphorylation sites, such as AT8 [35] and p-S422 [31].

One important aspect of our study is the strategy used to validate in vitro findings in an in vivo system easily amenable to study cell division within an epithelial layer after three days of transient human Tau overexpression. We chose *Drosophila melanogaster* because this model proved useful to study human Tau phosphorylation in the nervous tissue [44,45] and because the molecular mechanisms governing cell division are evolutionarily conserved across animal species [46,47]. This model also enabled to validate the observed phospho-signature in non-cancerous cells. In this system, the phospho-epitopes AT8, p-T217, and p-S422 displayed clear and strong staining that was almost exclusively localized to mitotic cells, confirming their mitotic specificity. However, unlike the in vitro results, we observed distinct staining for p-T205 and p-S416 in non-mitotic cells. These findings are consistent with the increase in staining, upon Tau overexpression, observed during interphase under in vitro conditions: the fold increases in staining intensity for AT8, p-T217, and p-S422 were modest (1.42-fold, 1.48-fold, and 1.65-fold, respectively) compared to the more substantial increases observed for p-T205 and p-S416 (5.34-fold and 3.39-fold, respectively). This suggests that the slight increases in phosphorylation levels of p-T205 and p-S416 upon Tau overexpression during interphase in cell culture did not interfere with detecting additional phosphorylation at these sites during mitosis. However, in the *Drosophila* wing disc in vivo, this distinction was less apparent. Whether this observation applies broadly to other tissues warrants further investigation.

Our study focused on investigating the physiological phosphorylation of Tau during the cell cycle. This work contributes to a broader effort to understand Tau phosphorylation under physiological conditions, such as during development or in response to hibernation and fluctuations in body temperature [48,49,50]. Here, we demonstrate that p-T217, a recently identified biomarker of early AD, is highly phosphorylated during mitosis, alongside the previously characterized AT8 and p-S422 phospho-epitopes. These findings lend support to the longstanding hypothesis that cell-cycle reentry occurs in degenerating neurons during AD [51].

Critically, these results may also provide mechanistic insight into the emerging significance of p-T217 as an AD biomarker. Alongside the AT8 epitope, p-T217 is detected in post-mortem brains of both asymptomatic individuals (Braak stages 0–III) and symptomatic AD patients [52], suggesting that these phosphorylations occur early in disease progression, potentially during preclinical or mild cognitive impairment stages. Furthermore, recent studies highlight that plasma p-T217 exhibits superior diagnostic accuracy for AD compared to other established tau biomarkers [20,53,54].

Hence, specific kinases implicated in phosphorylating T217 as well as AT8 epitope and S422, their differential activity during mitosis—and their potential link to AD pathogenesis—warrants further investigation. Recent reviews [55,56] and the Hanger lab’s updated database (bit.ly/2JyZTbS) indicate that kinases known to phosphorylate both S202 and T205 for AT8 epitope, as well as T217 and S422 are GSK3, p38, ERK1/2, JNK1-3, and CK1/2. Some of these kinases (e.g., ERK) have emerging roles in G2/M transition or mitotic organelle distribution [57,58] or in mitotic checkpoint (e.g., CK1, GSK3) [59,60]. Previous work on recombinant Tau and Lan5 cell extracts showed that the mitotic cdk1/cdc2 kinase can phosphorylate Tau within a T212/T217 peptide [7]. To get further insight in the putative role of mitotic kinases like CDK1, Aurora, PLK and Nek [61], we used the GPS 6.0 software (https://gps.biocuckoo.cn/, accessed on 18 September 2025) to screen the 2N4R Tau sequence [62]. We found that S202, T205, T217, and S422 match CDK1 and Nek2 consensus motifs (Supplementary Table S3). None of these aligned with Aurora or PLK consensus sequences.

Our work focused on the 4R isoform, which is more prone to aggregation [63], and overrepresented in insoluble fibrils in AD post-mortem tissues compared to the 3R isoform [52]. While all phospho-sites tested are present in the 3R isoform, future studies should examine this isoform to determine whether the mitotic phospho-signature is isoform-dependent.

A key unresolved question concerns the functional consequences of this mitotic phosphorylation pattern on Tau’s cellular roles. The most immediate hypothesis is that it triggers Tau detachment from microtubules, a known mitotic event [7]. Given Tau’s established roles in promoting microtubule growth and inhibiting microtubule shrinkage [64,65], its release from microtubules could facilitate the dynamic microtubule remodeling essential for spindle assembly and mitotic progression. However, whether this specific phospho-signature directly impairs Tau–microtubule binding remains to be experimentally validated. Supporting this possibility, studies using a pseudo-phosphorylated TauE14 mutant—which mimics phosphorylation at all mitotic signature sites plus ten additional epitopes—demonstrate a dramatically reduced microtubule affinity [38,66].

Emerging evidence further suggests that modulating Tau phosphorylation in cancer cells can alter cell cycle progression [30]. Moving forward, it will be critical to dissect Tau’s functional contributions to mitotic microtubule dynamics and clarify how its phosphorylation state regulates these processes. Such work may bridge critical gaps in our understanding of Tau’s dual roles in normal cell division and neurodegenerative pathology.

## Figures and Tables

**Table 1:** Quantification of phospho-epitope immunoreactivity (IR) variations under different conditions (mitosis vs. interphase, Tau overexpression vs. basal) in Tau-inducible SH-SY5Y cells. Data are presented as mean ± SEM. Sample sizes: N = 2 biological replicates for each phospho-epitope, except AT8 and PHF1 (N = 3); total Tau staining (N = 8, with 4 datasets obtained using the DAKO antibody and 4 with the T46 antibody). Statistical analysis was performed using the Mann–Whitney non-parametric test to compare each phospho-epitope with total Tau staining, indicating significant differences. * *p* ≤ 0.05. Data for each replicate are detailed in Supplementary Table S1.

Epitope	IR Increase (Mitosis/Interphase) in Tau Overexpressing Cells	IR Increase (Overexpression of Tau/Basal Conditions) During Mitosis	IR Increase (Overexpression of Tau/Basal Conditions) During Interphase
AT8	60.52 ± 7.34 *	15.76 ± 2.80	1.42 ± 0.23 *
p-S202	0.43 ± 0.03 *	4.41 ± 1.66 *	19.11 ± 8.91
p-T205	27.32 ± 5.70 *	26.48 ± 3.04	5.34 ± 0.42 *
PHF1	3.95 ± 0.46 *	26.02 ± 6.61	20.37 ± 3.36
p-S396	3.10 ± 0.01 *	3.38 ± 0.20 *	8.99 ± 0.57
p-S404	1.21 ± 0.05 *	35.14 ± 1.75	30.41 ± 3.92
AT100	3.97 ± 1.05 *	0.75 ± 0.09 *	0.70 ± 0.13 *
p-T212	6.61 ± 0.76 *	2.41 ± 0.25 *	1.75 ± 0.32 *
p-S214	6.69 ± 0.12 *	1.42 ± 0.17 *	0.9 ± 0.09 *
p-T217	30.52 ± 13.25 *	8.0 ± 1.13	1.48 ± 0.65 *
p-S416	13.46 ± 1.93 *	17.62 ± 7.90	3.39 ± 1.67 *
p-S422	27.96 ± 2.82 *	28.17 ± 2.59	1.65 ± 0.41 *
Tot Tau	0.95 ± 0.03	17.41 ± 4.93	19.51 ± 4.76

## Data Availability

The original contributions presented in this study are included in the article/Supplementary Material. Further inquiries can be directed to the corresponding author.

## Supplementary Materials

The following supporting information can be downloaded at https://www.mdpi.com/article/10.3390/cells14201638/s1, Figure S1: cell cycle synchronization at interphase was achieved using a double thymidine block. Cells were first treated with 2 mM thymidine for 18 h, followed by a 10-h release period. A second thymidine treatment was then applied to enhance the homogeneity of cells arrested in interphase. (A) Following the second release, cells resumed synchronized progression through the cell cycle, with a mitotic peak observed approximately 12 h later (T12) as assessed by PH3. (B) Total Tau and phospho-Tau staining show an increase in AT8, pT217, pS416 and pS422 epitopes at the mitotic peak (12 h) compared to 0 h after thymidine released. Total Tau staining is unchanged throughout the cell cycle; Table S1: Original data used for Table 1; Table S2: Data corresponding to graphs shown in Figure 1 and Figure 2. Table S3A: Raw results from search of consensus sequences for specified kinases with GPS6.0. Table S3B: Summary of consensus phosphorylation sites identified at each studied Tau residue.

## References

[1] Weingarten M.D., Lockwood A.H., Hwo S.Y., Kirschner M.W. (1975). A Protein Factor Essential for Microtubule Assembly. Proc. Natl. Acad. Sci. USA.

[2] Witman G.B., Cleveland D.W., Weingarten M.D., Kirschner M.W. (1976). Tubulin Requires Tau for Growth onto Microtubule Initiating Sites. Proc. Natl. Acad. Sci. USA.

[3] Barbier P., Zejneli O., Martinho M., Lasorsa A., Belle V., Smet-Nocca C., Tsvetkov P.O., Devred F., Landrieu I. (2019). Role of Tau as a Microtubule-Associated Protein: Structural and Functional Aspects. Front. Aging Neurosci..

[4] Biernat J., Gustke N., Drewes G., Mandelkow E.M., Mandelkow E. (1993). Phosphorylation of Ser262 Strongly Reduces Binding of Tau to Microtubules: Distinction between PHF-like Immunoreactivity and Microtubule Binding. Neuron.

[5] Scott C.W., Spreen R.C., Herman J.L., Chow F.P., Davison M.D., Young J., Caputo C.B. (1993). Phosphorylation of Recombinant Tau by cAMP-Dependent Protein Kinase. Identification of Phosphorylation Sites and Effect on Microtubule Assembly. J. Biol. Chem..

[6] Sengupta A., Kabat J., Novak M., Wu Q., Grundke-Iqbal I., Iqbal K. (1998). Phosphorylation of Tau at Both Thr 231 and Ser 262 Is Required for Maximal Inhibition of Its Binding to Microtubules. Arch. Biochem. Biophys..

[7] Illenberger S., Zheng-Fischhöfer Q., Preuss U., Stamer K., Baumann K., Trinczek B., Biernat J., Godemann R., Mandelkow E.-M., Mandelkow E. (1998). The Endogenous and Cell Cycle-Dependent Phosphorylation of Tau Protein in Living Cells: Implications for Alzheimer’s Disease. Mol. Biol. Cell.

[8] Schneider A., Biernat J., Von Bergen M., Mandelkow E., Mandelkow E.-M. (1999). Phosphorylation That Detaches Tau Protein from Microtubules (Ser262, Ser214) Also Protects It against Aggregation into Alzheimer Paired Helical Filaments. Biochemistry.

[9] Evans D.B., Rank K.B., Bhattacharya K., Thomsen D.R., Gurney M.E., Sharma S.K. (2000). Tau Phosphorylation at Serine 396 and Serine 404 by Human Recombinant Tau Protein Kinase II Inhibits Tau’s Ability to Promote Microtubule Assembly. J. Biol. Chem..

[10] Otvos L., Feiner L., Lang E., Szendrei G.I., Goedert M., Lee V.M. (1994). Monoclonal Antibody PHF-1 Recognizes Tau Protein Phosphorylated at Serine Residues 396 and 404. J. Neurosci. Res..

[11] Wegmann S., Biernat J., Mandelkow E. (2021). A Current View on Tau Protein Phosphorylation in Alzheimer’s Disease. Curr. Opin. Neurobiol..

[12] Alonso A.D.C., Zaidi T., Novak M., Grundke-Iqbal I., Iqbal K. (2001). Hyperphosphorylation Induces Self-Assembly of τ into Tangles of Paired Helical Filaments/Straight Filaments. Proc. Natl. Acad. Sci. USA.

[13] Buée L., Bussière T., Buée-Scherrer V., Delacourte A., Hof P.R. (2000). Tau Protein Isoforms, Phosphorylation and Role in Neurodegenerative disorders11These Authors Contributed Equally to This Work. Brain Res. Rev..

[14] Despres C., Byrne C., Qi H., Cantrelle F.-X., Huvent I., Chambraud B., Baulieu E.-E., Jacquot Y., Landrieu I., Lippens G. (2017). Identification of the Tau Phosphorylation Pattern That Drives Its Aggregation. Proc. Natl. Acad. Sci. USA.

[15] Luna-Muñoz J., Chávez-Macías L., García-Sierra F., Mena R. (2007). Earliest Stages of Tau Conformational Changes Are Related to the Appearance of a Sequence of Specific Phospho-Dependent Tau Epitopes in Alzheimer’s Disease1. J. Alzheimer’s Dis..

[16] Augustinack J.C., Sanders J.L., Tsai L.-H., Hyman B.T. (2002). Colocalization and Fluorescence Resonance Energy Transfer between Cdk5 and AT8 Suggests a Close Association in Pre-Neurofibrillary Tangles and Neurofibrillary Tangles. J. Neuropathol. Exp. Neurol..

[17] Goedert M., Jakes R., Vanmechelen E. (1995). Monoclonal Antibody AT8 Recognises Tau Protein Phosphorylated at Both Serine 202 and Threonine 205. Neurosci. Lett..

[18] Barthélemy N.R., Li Y., Janelidze S., He Y., Xiong C., Stomrud E., Fagan A.M., Karch C.M., Benzinger T.L.S., McDade E. (2023). Plasma Tau Phosphorylation at T217 Predicts Amyloid Deposition in Dominantly Inherited and Late Onset Alzheimer Disease Participants without Clinical Symptoms. Alzheimer’s Dement..

[19] Barthélemy N.R., Bateman R.J., Hirtz C., Marin P., Becher F., Sato C., Gabelle A., Lehmann S. (2020). Cerebrospinal Fluid Phospho-Tau T217 Outperforms T181 as a Biomarker for the Differential Diagnosis of Alzheimer’s Disease and PET Amyloid-Positive Patient Identification. Alzheimer’s Res. Ther..

[20] Grande G., Valletta M., Rizzuto D., Xia X., Qiu C., Orsini N., Dale M., Andersson S., Fredolini C., Winblad B. (2025). Blood-Based Biomarkers of Alzheimer’s Disease and Incident Dementia in the Community. Nat. Med..

[21] Janelidze S., Stomrud E., Smith R., Palmqvist S., Mattsson N., Airey D.C., Proctor N.K., Chai X., Shcherbinin S., Sims J.R. (2020). Cerebrospinal Fluid P-Tau217 Performs Better than p-Tau181 as a Biomarker of Alzheimer’s Disease. Nat. Commun..

[22] Kanaan N.M., Cox K., Alvarez V.E., Stein T.D., Poncil S., McKee A.C. (2016). Characterization of Early Pathological Tau Conformations and Phosphorylation in Chronic Traumatic Encephalopathy. J. Neuropathol. Exp. Neurol..

[23] Voss K., Koren J., Dickey C.A. (2011). The Earliest Tau Dysfunction in Alzheimer’s Disease?. Am. J. Pathol..

[24] Balczon R., Lin M.T., Lee J.Y., Abbasi A., Renema P., Voth S.B., Zhou C., Koloteva A., Michael Francis C., Sodha N.R. (2021). Pneumonia Initiates a Tauopathy. FASEB J..

[25] Barker R.M., Chambers A., Kehoe P.G., Rowe E., Perks C.M. (2024). Untangling the Role of Tau in Sex Hormone Responsive Cancers: Lessons Learnt from Alzheimer’s Disease. Clin. Sci..

[26] Chapelet G., Béguin N., Castellano B., Grit I., De Coppet P., Oullier T., Neunlist M., Blottière H., Rolli-Derkinderen M., Le Dréan G. (2023). Tau Expression and Phosphorylation in Enteroendocrine Cells. Front. Neurosci..

[27] Gargini R., Segura-Collar B., Sánchez-Gómez P. (2019). Novel Functions of the Neurodegenerative-Related Gene Tau in Cancer. Front. Aging Neurosci..

[28] Sigala J., Jumeau F., Buée L., Sergeant N., Mitchell V. (2015). La protéine microtubulaire Tau testiculaire: Une place dans la spermatogenèse?. Morphologie.

[29] Vanier M.-T., Neuville P., Michalik L., Launay J.-F. (1998). Expression of Specific Tau Exons in Normal and Tumoral Pancreatic Acinar Cells. J. Cell Sci..

[30] Clementi L., Sabetta S., Zelli V., Compagnoni C., Tessitore A., Mattei V., Angelucci A. (2023). Mitotic Phosphorylation of Tau/MAPT Modulates Cell Cycle Progression in Prostate Cancer Cells. J. Cancer Res. Clin. Oncol..

[31] Delobel P., Flament S., Hamdane M., Mailliot C., Sambo A., Bégard S., Sergeant N., Delacourte A., Vilain J., Buée L. (2002). Abnormal Tau Phosphorylation of the Alzheimer-type Also Occurs during Mitosis. J. Neurochem..

[32] Hamdane M., Sambo A.-V., Delobel P., Bégard S., Violleau A., Delacourte A., Bertrand P., Benavides J., Buée L. (2003). Mitotic-like Tau Phosphorylation by P25-Cdk5 Kinase Complex. J. Biol. Chem..

[33] Pope W.B., Lambert M.P., Leypold B., Seupaul R., Sletten L., Krafft G., Klein W.L. (1994). Microtubule-Associated Protein Tau Is Hyperphosphorylated during Mitosis in the Human Neuroblastoma Cell Line SH-SY5Y. Exp. Neurol..

[34] Preuss U., Döring F., Illenberger S., Mandelkow E.M. (1995). Cell Cycle-Dependent Phosphorylation and Microtubule Binding of Tau Protein Stably Transfected into Chinese Hamster Ovary Cells. Mol. Biol. Cell.

[35] Preuss U., Mandelkow E.-M. (1998). Mitotic Phosphorylation of Tau Protein in Neuronal Cell Lines Resembles Phosphorylation in Alzheimer’s Disease. Eur. J. Cell Biol..

[36] Bretteville A., Ando K., Ghestem A., Loyens A., Bégard S., Beauvillain J.-C., Sergeant N., Hamdane M., Buée L. (2009). Two-Dimensional Electrophoresis of Tau Mutants Reveals Specific Phosphorylation Pattern Likely Linked to Early Tau Conformational Changes. PLoS ONE.

[37] Bougé A.-L., Parmentier M.-L. (2016). Tau Excess Impairs Mitosis and Kinesin-5 Function, Leading to Aneuploidy and Cell Death. Dis. Models Mech..

[38] Fulga T.A., Elson-Schwab I., Khurana V., Steinhilb M.L., Spires T.L., Hyman B.T., Feany M.B. (2007). Abnormal Bundling and Accumulation of F-Actin Mediates Tau-Induced Neuronal Degeneration in Vivo. Nat. Cell Biol..

[39] Schindelin J., Arganda-Carreras I., Frise E., Kaynig V., Longair M., Pietzsch T., Preibisch S., Rueden C., Saalfeld S., Schmid B. (2012). Fiji: An Open-Source Platform for Biological-Image Analysis. Nat. Methods.

[40] Yoshida H., Goedert M. (2006). Sequential Phosphorylation of Tau Protein by cAMP-dependent Protein Kinase and SAPK4/P38δ or JNK2 in the Presence of Heparin Generates the AT100 Epitope. J. Neurochem..

[41] Nadel C.M., Pokhrel S., Wucherer K., Oehler A., Thwin A.C., Basu K., Callahan M.D., Southworth D.R., Mordes D.A., Craik C.S. (2024). Phosphorylation of Tau at a Single Residue Inhibits Binding to the E3 Ubiquitin Ligase, CHIP. Nat. Commun..

[42] Denus M., Filaquier A., Fargues W., Néel E., Stewart S.E., Colladant M., Curel T., Mezghrani A., Marin P., Claeysen S. (2025). A Sensitive and Versatile Cell-Based Assay Combines Luminescence and Trapping Approaches to Monitor Unconventional Protein Secretion. Traffic.

[43] Curel T., Denus M., Villeneuve J., Parmentier M.-L. (2025). A New in Vivo Model of Human Tau Excess with Sustained Activation of Caspases.

[44] Jackson G.R., Wiedau-Pazos M., Sang T.-K., Wagle N., Brown C.A., Massachi S., Geschwind D.H. (2002). Human Wild-Type Tau Interacts with Wingless Pathway Components and Produces Neurofibrillary Pathology in Drosophila. Neuron.

[45] Steinhilb M.L., Dias-Santagata D., Fulga T.A., Felch D.L., Feany M.B. (2007). Tau Phosphorylation Sites Work in Concert to Promote Neurotoxicity In Vivo. Mol. Biol. Cell.

[46] Cross F.R., Buchler N.E., Skotheim J.M. (2011). Evolution of Networks and Sequences in Eukaryotic Cell Cycle Control. Phil. Trans. R. Soc. B.

[47] Glover D.M. (1989). Mitosis in *Drosophila*. J. Cell Sci..

[48] Canet G., Rocaboy E., Laliberté F., Boscher E., Guisle I., Diego-Diaz S., Fereydouni-Forouzandeh P., Whittington R.A., Hébert S.S., Pernet V. (2023). Temperature-Induced Artifacts in Tau Phosphorylation: Implications for Reliable Alzheimer’s Disease Research. Exp. Neurobiol..

[49] Duquette A., Pernègre C., Veilleux Carpentier A., Leclerc N. (2021). Similarities and Differences in the Pattern of Tau Hyperphosphorylation in Physiological and Pathological Conditions: Impacts on the Elaboration of Therapies to Prevent Tau Pathology. Front. Neurol..

[50] León-Espinosa G., Murillo A.M.M., Turegano-Lopez M., DeFelipe J., Holgado M. (2024). Phosphorylated Tau at T181 Accumulates in the Serum of Hibernating Syrian Hamsters and Rapidly Disappears after Arousal. Sci. Rep..

[51] Barrio-Alonso E., Hernández-Vivanco A., Walton C.C., Perea G., Frade J.M. (2018). Cell Cycle Reentry Triggers Hyperploidization and Synaptic Dysfunction Followed by Delayed Cell Death in Differentiated Cortical Neurons. Sci. Rep..

[52] Wesseling H., Mair W., Kumar M., Schlaffner C.N., Tang S., Beerepoot P., Fatou B., Guise A.J., Cheng L., Takeda S. (2020). Tau PTM Profiles Identify Patient Heterogeneity and Stages of Alzheimer’s Disease. Cell.

[53] Warmenhoven N., Salvadó G., Janelidze S., Mattsson-Carlgren N., Bali D., Dolado A.O., Kolb H., Triana-Baltzer G., Barthélemy N.R., Schindler S.E. (2024). A Comprehensive Head-to-Head Comparison of Key Plasma Phosphorylated Tau 217 Biomarker Tests. medRxiv.

[54] Lai R., Li B., Bishnoi R. (2024). P-Tau217 as a Reliable Blood-Based Marker of Alzheimer’s Disease. Biomedicines.

[55] Montalto G., Ricciarelli R. (2023). Tau, Tau Kinases, and Tauopathies: An Updated Overview. BioFactors.

[56] Martin L., Latypova X., Wilson C.M., Magnaudeix A., Perrin M.-L., Yardin C., Terro F. (2013). Tau Protein Kinases: Involvement in Alzheimer’s Disease. Ageing Res. Rev..

[57] Shaul Y.D., Seger R. (2006). ERK1c Regulates Golgi Fragmentation during Mitosis. J. Cell Biol..

[58] Shapiro P.S., Vaisberg E., Hunt A.J., Tolwinski N.S., Whalen A.M., McIntosh J.R., Ahn N.G. (1998). Activation of the MKK/ERK Pathway during Somatic Cell Mitosis: Direct Interactions of Active ERK with Kinetochores and Regulation of the Mitotic 3F3/2 Phosphoantigen. J. Cell Biol..

[59] Johnson A.E., Chen J.-S., Gould K.L. (2013). CK1 Is Required for a Mitotic Checkpoint That Delays Cytokinesis. Curr. Biol..

[60] Rashid M.S., Mazur T., Ji W., Liu S.T., Taylor W.R. (2018). Analysis of the Role of GSK3 in the Mitotic Checkpoint. Sci. Rep..

[61] Salaun P., Rannou Y., Claude P., Li J.J., Li S.A., Mohla S., Rochefort H., Maudelonde T. (2008). Cdk1, Plks, Auroras, and Neks: The Mitotic Bodyguards. Hormonal Carcinogenesis V..

[62] Chen M., Zhang W., Gou Y., Xu D., Wei Y., Liu D., Han C., Huang X., Li C., Ning W. (2023). GPS 6.0: An Updated Server for Prediction of Kinase-Specific Phosphorylation Sites in Proteins. Nucleic Acids Res..

[63] Zhong Q., Congdon E.E., Nagaraja H.N., Kuret J. (2012). Tau Isoform Composition Influences Rate and Extent of Filament Formation. J. Biol. Chem..

[64] Prezel E., Elie A., Delaroche J., Stoppin-Mellet V., Bosc C., Serre L., Fourest-Lieuvin A., Andrieux A., Vantard M., Arnal I. (2018). Tau Can Switch Microtubule Network Organizations: From Random Networks to Dynamic and Stable Bundles. Mol. Biol. Cell.

[65] Noble W., Hanger D.P., Miller C.C.J., Lovestone S. (2013). The Importance of Tau Phosphorylation for Neurodegenerative Diseases. Front. Neurol..

[66] Talmat-Amar Y., Arribat Y., Parmentier M.-L. (2018). Vesicular Axonal Transport Is Modified In Vivo by Tau Deletion or Overexpression in Drosophila. Int. J. Mol. Sci..

