# Supplementary material for: Phospho-Tau Signature During Mitosis: AT8, p-T217 and p-S422 as Key Phospho-Epitopes
Source: Cells. 2025 Oct 21;14(20):1638. doi: 10.3390/cells14201638 (PMC12562719; doi:10.3390/cells14201638)
Supplement: Supplementary file 1 [file cells-14-01638-s001.zip › Supplementary Fig1 new2.pdf]

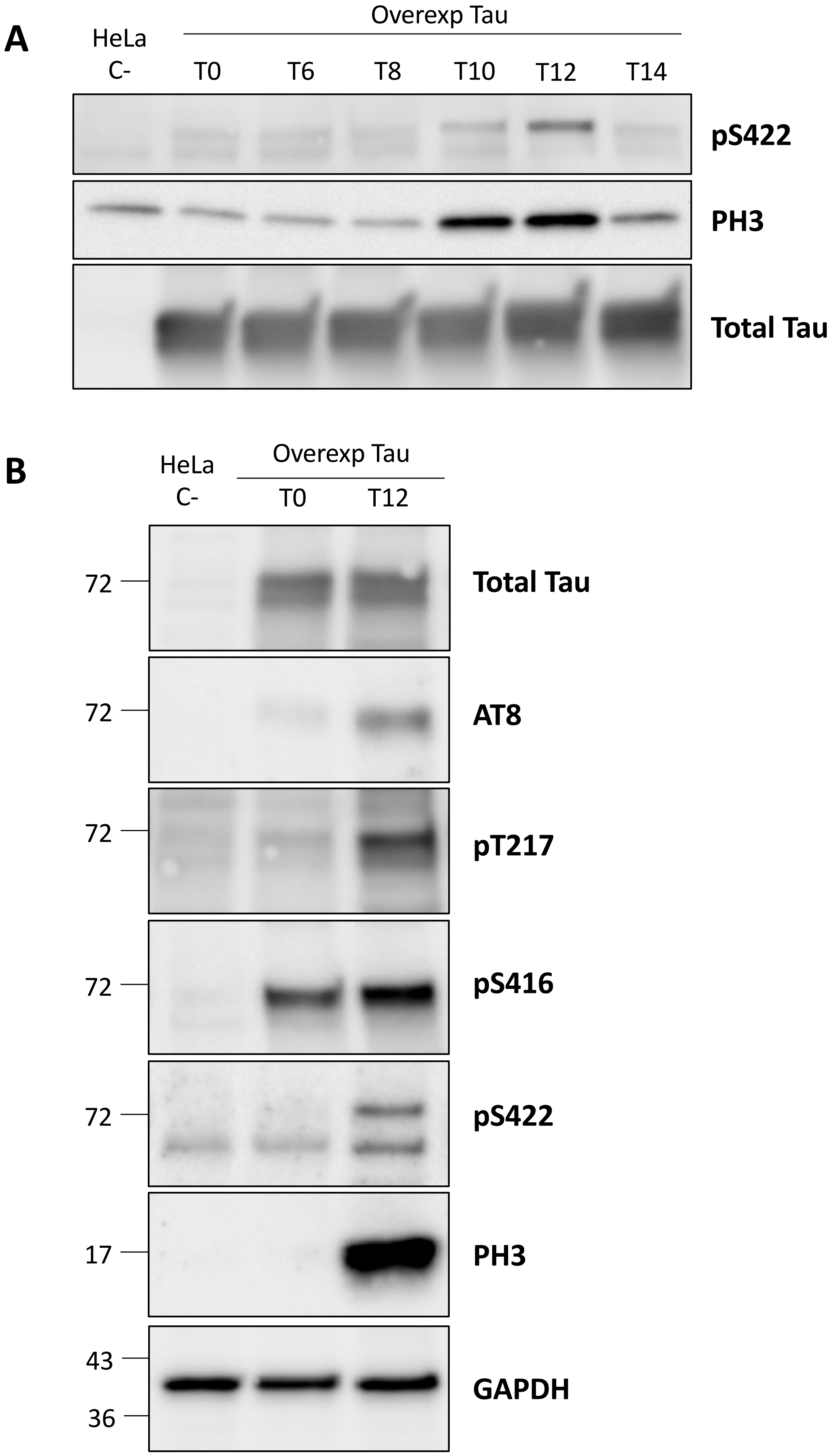

Legend: cell cycle synchronization at interphase was achieved using a double thymidine block. Cells were first treated with 2 mM thymidine for 18 hours, followed by a 10-hour release period. A second thymidine treatment was then applied to enhance the homogeneity of cells arrested in interphase. A) Following the second release, cells resumed synchronized progression through the cell cycle, with a mitotic peak observed approximately 12 hours later (T12) as assessed by PH3. B) Total Tau and phospho-Tau staining show an increase in AT8, pT217, pS416 and pS422 epitopes at the mitotic peak (12h) compared to 0h after thymidine released. Total Tau staining is unchanged throughout the cell cycle.

Sup. Fig. S1
