# Supplementary material for: Phospho-Tau Signature During Mitosis: AT8, p-T217 and p-S422 as Key Phospho-Epitopes
Source: Cells. 2025 Oct 21;14(20):1638. doi: 10.3390/cells14201638 (PMC12562719; doi:10.3390/cells14201638)
Supplement: Supplementary file 1 [file cells-14-01638-s001.zip › Supplementary material cells.pdf]

Supplementary material:

Stably transfected HeLa cell line with 0N4R Tau construct.

To generate HeLa cell line stably expressing Tau, a plasmid with 0N4R Tau sequence fused to the HiBit and SBP, as described in [1] was transfected using TransIT-2020 transfection reagent (Mirus, #MIR5400), according to the manufacturer's recommendations. After 2 days, transfected cells were selected with 600 µg/mL G-418 (Sigma, #4727878001) for 1 week. GFP-positive cells were then isolated by fluorescence-activated cell sorting (FACS). After expansion into a six-well plate format, the cell lines were assessed by Western blot analysis.

Cell cycle synchronization protocol

HeLa cells line (ATCC #CCL-2; CVCL_0030) was grown in complete culture medium consisting of DMEM (Sigma, #D8437) supplemented with 10% heat-inactivated FBS (Gibco, #10270-106), 100 U/mL penicillin, 100 µg/mL streptomycin (Gibco, #15140-122) and 2 mM l-glutamine (Sigma, #G7513) and maintained in a humidified atmosphere containing 5% CO2 at 37°C. Stably Tau-expressing HeLa cells were synchronized using a double thymidine block. Cells underwent an initial 18-hour treatment with 2 mM thymidine, followed by a 10-hour release period. A second thymidine treatment was then applied to improve synchronization homogeneity of interphase-arrested cells. Following the second release, cells progressed synchronously through the cell cycle, with a mitotic peak observed at ~12 hours. Cell extracts were obtained at different time points after the second release.

Western Blot protocol

Cells were lysed for 30min on ice in lysis buffer (20mM Tris, pH 7.4, 150 mM NaCl, 2mM EDTA, 2mM EGTA, 2% [v/v] Triton X-100) supplemented with a protease inhibitor cocktail (Sigma, #11836170001), 1mM Na_3VO_4, 10mM sodium fluoride, 5mM $Na_4P_2O_7$, 50mM B-Glycerophosphate, and centrifuged at 16000 g for 15min

Samples were incubated with 1X SDS loading buffer at 95°C for 10 minutes, then separated by SDS-PAGE electrophoresis (4–20% gradient, Mini-PROTEAN TGX Stain-Free gel). Proteins were subsequently transferred onto a nitrocellulose membrane.

After transfer, membranes were blocked with 5% BSA in PBS containing 0.1% Tween-20 (PBS-T) for 1 hour at room temperature, followed by overnight incubation at 4°C with primary antibodies diluted in PBS-T with 5% BSA. After three 10-minute washes in PBS-T, membranes were incubated for 1 hour at room temperature with HRP-conjugated secondary antibodies in PBS-T with 5% BSA. For For PH3, PSD95, and β3-tubulin antibodies, all steps were performed in PBS-T containing 5% milk.

Immunoreactive bands were detected using enhanced chemiluminescence (ECL) (Western Blotting Detection Reagent, Bio-Rad #170-5061 or GeneTex #GTX14698).
