# Supplementary material for: Phospho-Tau Signature During Mitosis: AT8, p-T217 and p-S422 as Key Phospho-Epitopes
Source: Cells. 2025 Oct 21;14(20):1638. doi: 10.3390/cells14201638 (PMC12562719; doi:10.3390/cells14201638)
Supplement: Supplementary file 1 [file cells-14-01638-s001.zip › Supplementary Table S1.pdf]

Sup. Table S1: original data used for Table 1

epitope	replicate number	effect mitosis in Tau OE conditions	effect Tau OE in mitotic cells	effect Tau OE in interphase cells
AT8	repl1	81	25.38	0.63
AT8	repl2	63.32	11.87	1.96
AT8	repl3	37.23	10.03	1.67
p-S202	repl1	0.39	2.07	6.51
p-S202	repl2	0.47	6.76	31.705
p-T205	repl1	35.39	30.79	5.94
p-T205	repl2	19.26	22.18	4.75
PHF1	repl1	3.11	26.78	24.78
PHF1	repl2	3.18	5.8	8.83
PHF1	repl3	5.55	45.48	27.51
p-S396	repl1	3.1	3.66	9.8
p-S396	repl2	3.1	3.1	8.18
p-S404	repl1	1.15	37.62	35.96
p-S404	repl2	1.28	32.66	24.86
AT100	repl1	5.45	0.63	0.52
AT100	repl2	2.49	0.88	0.89
p-T212	repl1	7.68	2.06	1.3
p-T212	repl2	5.54	2.76	2.2
p-S214	repl1	6.86	1.66	1.03
p-S214	repl2	6.52	1.19	0.77
p-T217	repl1	11.78	6.4	2.4
p-T217	repl2	49.27	9.6	0.56
p-S416	repl1	16.19	28.8	5.76
p-S416	repl2	10.73	6.45	1.03
p-S422	repl1	31.95	31.83	2.24
p-S422	repl2	23.97	24.51	1.07
p-S422	repl3	337.25	207.6	2.5
Total Tau (DAKO)	repl1	0.86	9.1	14.11
Total Tau (DAKO)	repl2	0.88	8.13	7.3
Total Tau (DAKO)	repl3	0.98	25.71	30.95
Total Tau (DAKO)	repl4	0.94	14.38	13.78
Total Tau (T46)	repl1	1.01	48.47	47.28
Total Tau (T46)	repl2	0.9	9.58	13.9
Total Tau (T46)	repl3	0.9	8.84	12.27
Total Tau (T46)	repl4	1.1	12.04	10.78
