# Supplementary material for: Phospho-Tau Signature During Mitosis: AT8, p-T217 and p-S422 as Key Phospho-Epitopes
Source: Cells. 2025 Oct 21;14(20):1638. doi: 10.3390/cells14201638 (PMC12562719; doi:10.3390/cells14201638)
Supplement: Supplementary file 1 [file cells-14-01638-s001.zip › Supplementary Table S2.pdf]

Sup Table S2: data corresponding to graphs shown in Fig. 1 and 2.

data graphs Fig. 1

total Tau (DAKO)

no tet		interphase		mitosis	with tet		interphase		mitosis
image 1	cell 1	100	cell 1	194	image 1	cell 1	1449	cell 1	2290
image 1	cell 2	175	cell 2	164	image 1	cell 2	1704	cell 2	1495
image 1	cell 3	166	cell 3	65	image 1	cell 3	2297	cell 3	2228
image 1	cell 4	92	cell 4	19	image 1	cell 4	2085	cell 4	1723
image 2	cell 5	168	cell 5	271	image 2	cell 5	1666	cell 5	1895
image 2	cell 6	132	cell 6	116	image 2	cell 6	1096	cell 6	1936
image 2	cell 7	332	cell 7	123	image 2	cell 7	1780	cell 7	755
image 2	cell 8	111	cell 8	111	image 2	cell 8	2298	cell 8	1307
image 3	cell 9	94	cell 9	86	image 3	cell 9	2850	cell 9	1950
image 3	cell 10	89	cell 10	85	image 3	cell 10	2081	cell 10	1233
image 3	cell 11	53	cell 11	90	image 3	cell 11	870	cell 11	2245
image 3	cell 12	80	cell 12	114					

AT8

no tet		interphase		mitosis	with tet		interphase		mitosis
image 1	cell 1	8	cell 1	279	image 1	cell 1	32	cell 1	2718
image 1	cell 2	8	cell 2	198	image 1	cell 2	36	cell 2	1751
image 1	cell 3	44	cell 3	147	image 1	cell 3	18	cell 3	2339
image 1	cell 4	9	cell 4	47	image 1	cell 4	138	cell 4	2330
image 2	cell 5	10	cell 5	305	image 2	cell 5	43	cell 5	1027
image 2	cell 6	5	cell 6	164	image 2	cell 6	20	cell 6	3708
image 2	cell 7	36	cell 7	219	image 2	cell 7	28	cell 7	1163
image 2	cell 8	12	cell 8	262	image 2	cell 8	28	cell 8	2294
image 3	cell 9	17	cell 9	356	image 3	cell 9	37	cell 9	3567

image 3	cell 10	58	cell 10	134		image 3	cell 10	29	cell 10	2099	
image 3	cell 11	5	cell 11	166		image 3	cell 11	8	cell 11	3752	
image 3	cell 12	14	cell 12	174							
p-S202											
no tet		interphase		mitosis		with tet		interphase		mitosis	
image 1	cell 1	175	cell 1	166		image 1	cell 1	753	cell 1	317	
image 1	cell 2	131	cell 2	243		image 1	cell 2	1110	cell 2	378	
image 1	cell 3	164	cell 3	196		image 1	cell 3	619	cell 3	383	
image 1	cell 4	154	cell 4	115		image 1	cell 4	491	cell 4	200	
image 2	cell 5	228	cell 5	264		image 2	cell 5	2142	cell 5	888	
image 2	cell 6	296	cell 6	336		image 2	cell 6	2308	cell 6	1499	
image 2	cell 7	109	cell 7	248		image 2	cell 7	1507	cell 7	547	
image 2	cell 8	150	cell 8	283		image 2	cell 8	1334	cell 8	231	
image 3	cell 9	289	cell 9	272		image 3	cell 9	1142	cell 9	468	
image 3	cell 10	175	cell 10	293		image 3	cell 10	1335	cell 10	459	
image 3	cell 11	206	cell 11	245		image 3	cell 11	892	cell 11	403	
image 3	cell 12	291	cell 12	251		image 3	cell 12	1816	cell 12	268	
p-T205											
no tet		interphase		mitosis		with tet		interphase		mitosis	
image 1	cell 1	7	cell 1	89		image 1	cell 1	70	cell 1	2331	
image 1	cell 2	35	cell 2	69		image 1	cell 2	17	cell 2	1185	
image 1	cell 3	5	cell 3	58		image 1	cell 3	16	cell 3	1625	
image 1	cell 4	6	cell 4	62		image 1	cell 4	69	cell 4	2581	
image 2	cell 5	4	cell 5	41		image 2	cell 5	70	cell 5	2524	
image 2	cell 6	7	cell 6	42		image 2	cell 6	19	cell 6	1885	
image 2	cell 7	2	cell 7	44		image 2	cell 7	21	cell 7	798	
image 2	cell 8	2	cell 8	77		image 2	cell 8	125	cell 8	1251	
image 3	cell 9	11	cell 9	34		image 3	cell 9	12	cell 9	1993	

image 3	cell 10	7	cell 10	52
image 3	cell 11	2	cell 11	57
image 3	cell 12	4	cell 12	46

image 3	cell 10	42	cell 10	1191
image 3	cell 11	78	cell 11	1969
image 3	cell 12	42	cell 12	1545

PHF1

no tet		interphase		mitosis
image 1	cell 1	18	cell 1	67
image 1	cell 2	13	cell 2	47
image 1	cell 3	21	cell 3	63
image 1	cell 4	17	cell 4	34
image 2	cell 5	17	cell 5	50
image 2	cell 6	17	cell 6	73
image 2	cell 7	12	cell 7	77
image 2	cell 8	20	cell 8	54

with tet		interphase		mitosis
image 1	cell 1	670	cell 1	2516
image 1	cell 2	147	cell 2	2576
image 1	cell 3	304	cell 3	1483
image 1	cell 4	502	cell 4	1922
image 2	cell 5	286	cell 5	3665
image 2	cell 6	586	cell 6	3145
image 2	cell 7	407	cell 7	3626
image 2	cell 8	730	cell 8	1202
image 3	cell 9	872	cell 9	3924
image 3	cell 10	301		

p-S396

no tet		interphase		mitosis
image 1	cell 1	148	cell 1	363
image 1	cell 2	107	cell 2	337
image 1	cell 3	120	cell 3	477
image 1	cell 4	97	cell 4	976
image 2	cell 5	63	cell 5	667
image 2	cell 6	66	cell 6	656
image 2	cell 7	65	cell 7	684
image 2	cell 8	56	cell 8	746
image 3	cell 9	11	cell 9	613
image 3	cell 10	53	cell 10	649
image 3	cell 11	11	cell 11	562

with tet		interphase		mitosis
image 1	cell 1	589	cell 1	1497
image 1	cell 2	374	cell 2	2996
image 1	cell 3	549	cell 3	1406
image 1	cell 4	1139	cell 4	978
image 2	cell 5	433	cell 5	2796
image 2	cell 6	430	cell 6	2222
image 2	cell 7	1256	cell 7	2157
image 2	cell 8	332	cell 8	2395
image 3	cell 9	891	cell 9	3408
image 3	cell 10	1266	cell 10	1670
image 3	cell 11	791	cell 11	3189

image 3	cell 12	73	cell 12	536	image 3	cell 12	544	cell 12	1946

p-S404

no tet		interphase		mitosis	with tet		interphase		mitosis
image 1	cell 1	29	cell 1	21	image 1	cell 1	795	cell 1	590
image 1	cell 2	22	cell 2	25	image 1	cell 2	494	cell 2	584
image 1	cell 3	32	cell 3	37	image 1	cell 3	464	cell 3	495
image 1	cell 4	39	cell 4	25	image 1	cell 4	394	cell 4	959
image 2	cell 5	15	cell 5	17	image 2	cell 5	1164	cell 5	1372
image 2	cell 6	17	cell 6	22	image 2	cell 6	272	cell 6	513
image 2	cell 7	15	cell 7	20	image 2	cell 7	614		
image 2	cell 8	18	cell 8	12	image 2				
image 3	cell 9	32	cell 9	40	image 3	cell 8	810	cell 7	2559
image 3	cell 10	27	cell 10	33	image 3	cell 9	1041	cell 8	720
image 3	cell 11	17	cell 11	37	image 3	cell 10	427	cell 9	1546
image 3	cell 12	22	cell 12	25	image 3	cell 11	3054	cell 10	677

data graphs Fig. 2

AT100

no tet		interphase		mitosis	with tet		interphase		mitosis
image 1	cell 1	699	cell 1	3634	image 1	cell 1	135	cell 1	1221
image 1	cell 2	508	cell 2	3335	image 1	cell 2	212	cell 2	2473
image 1	cell 3	404	cell 3	3126	image 1	cell 3	244	cell 3	1542
image 1					image 1	cell 4	344	cell 4	1502
image 2	cell 4	718	cell 4	3042	image 2	cell 5	583	cell 5	2229
image 2	cell 5	533	cell 5	3706	image 2	cell 6	356	cell 6	1788
image 2	cell 6	1277	cell 6	3913	image 2	cell 7	347	cell 7	2008
image 2					image 2	cell 8	177	cell 8	3152
image 3	cell 7	762	cell 7	2109	image 3	cell 9	1260	cell 9	2180

image 3	cell 8	620	cell 8	2435	image 3	cell 10	186	cell 10	2080
image 3	cell 9	679	cell 9	2375	image 3	cell 11	204	cell 11	1773
image 3	cell 10	855	cell 10	3453	image 3	cell 12	310	cell 12	1842
p-T212									
no tet		interphase		mitosis	with tet		interphase		mitosis
image 1	cell 1	102	cell 1	628	image 1	cell 1	84	cell 1	1423
image 1	cell 2	108	cell 2	626	image 1	cell 2	135	cell 2	1520
image 1	cell 3	76	cell 3	418	image 2	cell 3	97	cell 3	1592
image 1	cell 4	116	cell 4	632	image 2	cell 4	144	cell 4	2075
image 2	cell 5	414	cell 5	1039	image 2	cell 5	469	cell 5	1170
image 2	cell 6	134	cell 6	1005	image 3	cell 6	154	cell 6	1524
image 2	cell 7	251	cell 7	1211	image 3	cell 7	119	cell 7	1169
image 2	cell 8	184	cell 8	597	image 3	cell 8	138	cell 8	1291
image 3	cell 9	134	cell 9	844	image 3	cell 9	226	cell 9	1501
image 3	cell 10	79	cell 10	688	image 4	cell 10	665	cell 10	1445
image 3	cell 11	104	cell 11	855	image 4	cell 11	134	cell 11	2249
image 3	cell 12	162	cell 12	498	image 4	cell 12	69	cell 12	1766
p-S214									
no tet		interphase		mitosis	with tet		interphase		mitosis
image 1	cell 1	98	cell 1	448	image 1	cell 1	160	cell 1	2443
image 1	cell 2	294	cell 2	734	image 1	cell 2	136	cell 2	2655
image 1	cell 3	142	cell 3	1153	image 1	cell 3	227	cell 3	2125
image 1	cell 4	142	cell 4	789	image 1	cell 4	299	cell 4	1348
image 2	cell 5	355	cell 5	1497	image 2	cell 5	301	cell 5	1569
image 2	cell 6	123	cell 6	872	image 2	cell 6	180	cell 6	1001
image 2	cell 7	327	cell 7	992	image 2	cell 7	378	cell 7	1747
image 2	cell 8	386	cell 8	1494	image 2	cell 8	258		
image 3	cell 9	203	cell 9	1380	image 3	cell 9	205	cell 8	2458

image 3	cell 10	528	cell 10	1399

p-T217

no tet		interphase		mitosis
image 1	cell 1	115	cell 1	747
image 1	cell 2	96	cell 2	407
image 1	cell 3	51	cell 3	190
image 1	cell 4	98	cell 4	669
image 2	cell 5	176	cell 5	328
image 2	cell 6	66	cell 6	375
image 2	cell 7	115	cell 7	351
image 2	cell 8	65	cell 8	421

p-S416

no tet		interphase		mitosis
image 1	cell 1	35	cell 1	76
image 1	cell 2	17	cell 2	146
image 1	cell 3	26	cell 3	66
image 1	cell 4	40	cell 4	96
image 2	cell 5	25	cell 5	88
image 2	cell 6	27	cell 6	87
image 2	cell 7	28	cell 7	88
image 2	cell 8	21	cell 8	62

image 3	cell 10	400	cell 9	1047
image 3	cell 11	222	cell 10	1531
image 3	cell 12	360		

with tet		interphase		mitosis
image 1	cell 1	601	cell 1	3587
image 1	cell 2	354	cell 2	2634
image 1	cell 3	203	cell 3	3326
image 1	cell 4	640	cell 4	1782
image 2	cell 5	101	cell 5	2023
image 2	cell 6	137	cell 6	3082
image 2	cell 7	213	cell 7	3045
image 2	cell 8	218		
image 3	cell 9	191	cell 8	3980
image 3	cell 10	37	cell 9	1416
image 3	cell 11	67	cell 10	2848
image 3	cell 12	74	cell 11	2969

with tet		interphase		mitosis
image 1	cell 1	98	cell 1	454
image 1	cell 2	121	cell 2	4020
image 1	cell 3	210	cell 3	3920
image 1	cell 4	359	cell 4	4020
image 2	cell 5	138	cell 5	1374
image 2	cell 6	140	cell 6	1558
image 2	cell 7	144	cell 7	1900
image 2	cell 8	134	cell 8	1456
image 3	cell 9	176	cell 9	3060

image 3	cell 10	114	cell 10	3557
image 3	cell 11	82	cell 11	2519
image 3	cell 12	177	cell 12	2816

p-S422

no tet		interphase		mitosis
image 1	cell 1	23	cell 1	62
image 1	cell 2	43	cell 2	78
image 1	cell 3	38	cell 3	69
image 1	cell 4	22	cell 4	52
image 2	cell 5	27	cell 5	64
image 2	cell 6	52	cell 6	107
image 2	cell 7	14	cell 7	68
image 2	cell 8	25	cell 8	53

with tet		interphase		mitosis
image 1	cell 1	171	cell 1	691
image 1	cell 2	63	cell 2	1157
image 1	cell 3	56	cell 3	1228
image 1	cell 4	56	cell 4	2892
image 2	cell 5	84	cell 5	4023
image 2	cell 6	94	cell 6	3074
image 2	cell 7	33	cell 7	3427
image 2	cell 8	53		
image 3	cell 9	38	cell 8	1900
image 3	cell 10	41	cell 9	1565
