# Supplementary material for: Phospho-Tau Signature During Mitosis: AT8, p-T217 and p-S422 as Key Phospho-Epitopes
Source: Cells. 2025 Oct 21;14(20):1638. doi: 10.3390/cells14201638 (PMC12562719; doi:10.3390/cells14201638)
Supplement: Supplementary file 1 [file cells-14-01638-s001.zip › Supplementary Table S3.pdf]

Supplementary Table S3 :

Table A. Raw results from search of consensus sequences for specified kinases with GPS6.0

	ID	Position	Code	Kinase	PSP	Score	Cutoff (medium)
**NEK kinases**							
	Tau441	202	S	Other/NEK/NEK11	SGYSSPGSPGTPGSR	0.088	0.0537
	Tau441	202	S	Other/NEK/NEK1	SGYSSPGSPGTPGSR	0.3084	0.1182
	Tau441	205	T	Other/NEK/NEK1	SSPGSPGTPGSRSRT	0.1398	0.1182
	Tau441	217	T	Other/NEK/NEK1	SRTPSLPTPPTREPK	0.1664	0.1182
	Tau441	422	S	Other/NEK/NEK1	GSIDMVDSPQLATLA	0.2356	0.1182
	Tau441	202	S	Other/NEK/NEK2	SGYSSPGSPGTPGSR	0.5346	0.3454
	Tau441	205	T	Other/NEK/NEK2	SSPGSPGTPGSRSRT	0.5496	0.3454
	Tau441	217	T	Other/NEK/NEK2	SRTPSLPTPPTREPK	0.3942	0.3454
	Tau441	422	S	Other/NEK/NEK2	GSIDMVDSPQLATLA	0.695	0.3454
	Tau441	202	S	Other/NEK/NEK9	SGYSSPGSPGTPGSR	0.2151	0.1136
**CDK1**							
	Tau441	202	S	CMGC/CDK/CDC2/CDK1	SGYSSPGSPGTPGSR	0.1057	0.0262
	Tau441	205	T	CMGC/CDK/CDC2/CDK1	SSPGSPGTPGSRSRT	0.0836	0.0262
	Tau441	217	T	CMGC/CDK/CDC2/CDK1	SRTPSLPTPPTREPK	0.0599	0.0262
	Tau441	422	S	CMGC/CDK/CDC2/CDK1	GSIDMVDSPQLATLA	0.0426	0.0262
**Aurora kinases**							
	Tau441	no hit					
**Polo-like kinases**							
	Tau441	no hit					

Table B. Summary of consensus phosphorylation sites identified at each studied Tau residue

	NEK kinases				CDK1	Aurora	PLK
**S202**	NEK1	NEK2	NEK9	NEK11	CDK1		
**T205**	NEK1	NEK2			CDK1		
**T217**	NEK1	NEK2			CDK1		
**S422**	NEK1	NEK2			CDK1		

For each kinase, higher scores correspond to increased background color intensity.
